# Genetically-encoded tools for cAMP probing and modulation in living systems

**DOI:** 10.3389/fphar.2015.00196

**Published:** 2015-09-15

**Authors:** Valeriy M. Paramonov, Veronika Mamaeva, Cecilia Sahlgren, Adolfo Rivero-Müller

**Affiliations:** ^1^Department of Physiology, Institute of Biomedicine, University of Turku, Turku, Finland; ^2^Turku Center for Biotechnology, University of Turku and Åbo Akademi University, Turku, Finland; ^3^Department of Clinical Science, University of Bergen, Bergen, Norway; ^4^Department of Biomedical Engineering, Eindhoven University of Technology, Eindhoven, Netherlands; ^5^Faculty of Natural Sciences and Technology, Åbo Akademi University, Turku, Finland; ^6^Department of Biochemistry and Molecular Biology, Medical University of Lublin, Lublin, Poland

**Keywords:** cAMP signaling, cyclic nucleotide, biosensor, genetically encoded probe, optogenetics, cell-based assays

## Abstract

Intracellular 3′-5′-cyclic adenosine monophosphate (cAMP) is one of the principal second messengers downstream of a manifold of signal transduction pathways, including the ones triggered by G protein-coupled receptors. Not surprisingly, biochemical assays for cAMP have been instrumental for basic research and drug discovery for decades, providing insights into cellular physiology and guiding pharmaceutical industry. However, despite impressive track record, the majority of conventional biochemical tools for cAMP probing share the same fundamental shortcoming—all the measurements require sample disruption for cAMP liberation. This common bottleneck, together with inherently low spatial resolution of measurements (as cAMP is typically analyzed in lysates of thousands of cells), underpin the ensuing limitations of the conventional cAMP assays: (1) genuine kinetic measurements of cAMP levels over time in a single given sample are unfeasible; (2) inability to obtain precise information on cAMP spatial distribution and transfer at subcellular levels, let alone the attempts to pinpoint dynamic interactions of cAMP and its effectors. At the same time, tremendous progress in synthetic biology over the recent years culminated in drastic refinement of our toolbox, allowing us not only to bypass the limitations of conventional assays, but to put intracellular cAMP life-span under tight control—something, that seemed scarcely attainable before. In this review article we discuss the main classes of modern genetically-encoded tools tailored for cAMP probing and modulation in living systems. We examine the capabilities and weaknesses of these different tools in the context of their operational characteristics and applicability to various experimental set-ups involving living cells, providing the guidance for rational selection of the best tools for particular needs.

## Introduction

The small molecule 3′-5′-cyclic adenosine monophosphate (cAMP) is an established second messenger, involved in signal transduction in most living organisms. Being instrumental in regulation of a plethora of processes both in health and pathological states, principles of cAMP signaling have remained in the research spotlight for the best part of the last century and the interest to this cyclic nucleotide does not seem to fade away (Beavo and Brunton, 2002; Lefkimmiatis and Zaccolo, 2014). To a certain degree this is attributed to the immense pharmacological significance of G protein-coupled receptors (GPCRs), which have cAMP for an immediate intracellular effector and continue to serve as drug targets for as much as one third of the pharmaceutical compounds marketed worldwide (Filmore, 2004; Overington et al., 2006).

As it typically happens with branches of knowledge that are built up on empirical observations, the perceptions of cAMP-mediated signal transmission in living systems have been (and continue to be) shaped by the input from the experimental and analytical tools available to research community. The truism “*we can only go as far as our instruments will allow us to*” is nicely exemplified by the changes in the paradigm of cyclic nucleotide signaling, occurring in the last decades.

Conventional biochemical methods, such as chromatography with tritium—prelabeled adenine pool and antibody-dependent competition assays, that made the platform for cAMP studies till 1990-x, though robust and applicable to essentially any bodily tissue, can measure only the unresolved *bulk* of cAMP levels in pooled cellular populations, thus leaving us to guess what is going on with cAMP molecules in any given single cell. Apart from the limited spatial resolution, biochemical assays typically require cAMP liberation from specimens under study, which is usually accomplished by cell lysis (Williams, 2004; Hill et al., 2010). This way, biochemical assays in essence provide a single time point measurement, reflecting the total cAMP levels present in a specimen at the time of cell disruption. Though it is possible to deduce the overall kinetic trend of total cAMP over a time period by preparing a set of biological replicates and lysing them at certain intervals, the resulting kinetic curve is usually only a faint reflection of the actual cAMP oscillations in a given biological sample.

Experimental data on cAMP, obtained with biochemical methods with limited temporal and spatial resolution, formed the basis for a widely accepted model of cAMP signaling. This model implies cAMP generation by membrane-bound adenylyl cyclases (ACs) in response to GPCRs activation and its subsequent free diffusion into the cytoplasm. The ensuing activation of immediate cytoplasmic effectors of cAMP, such as protein kinase A (PKA), convey the signal further to the level of cell nucleus, eventually translating extracellular stimuli into transcriptional response (Beavo and Brunton, 2002).

However, cAMP network and governing principles of its functional and structural organization happen to be far more complex. Indeed, the conceptualization of cAMP signaling as of a highly compartmentalized process, occurring in separated subcellular domains, shaped by anchoring proteins and phosphodiesterases (PDEs), with organization of the key players of cAMP-mediated signal relay machinery into supramolecular complexes or “signalosomes,” has just started to evolve (Willoughby and Cooper, 2007; Lefkimmiatis and Zaccolo, 2014). Apart from the intricate laws of spatial organization of cAMP generation, trafficking and degradation, this burgeoning model recognizes the multifaceted nature of signal encoding by cAMP (strength vs. duration vs. frequency) and pays due regards to the crosstalk between cAMP and other intracellular regulators (Rich et al., 2014).

It wound not be an overstatement to say, that the major insights into the complexity of cAMP signaling, served to fuel the above conceptual framework, were gained by studies exploiting next generation of tools for cAMP probing and modulation. Most of these tools are genetically encoded proteins, tailored for sensing and modulation of cAMP in living systems. These engineered proteins provide excellent spatial resolution down to desired subcellular domains, can respond to genuine oscillations of cAMP levels in real time and are designed to uncover cAMP signaling partners, and as such have enabled a paradigm-shift in cyclic nucleotide research.

Evidently, in order to scrutinize a complex phenomenon, a set of diverse probing tools is required. Align with this and thanks to the intricate nature of cAMP signaling relay and never-ceasing attempts to gain insights into the *laws of the game*, several *families* of biosensors for cAMP have been developed (reviwed in Willoughby and Cooper, 2008; Hill et al., 2010; Sprenger and Nikolaev, 2013). However, besides being genetically-encoded proteins and hence applicable to studies in living cells, the modern biosensors do not have much in common, as they strive to probe different aspects of cAMP signaling, are governed by distinct biological phenomena and rely on diverse biophysical techniques.

Considering this heterogeneity and in order to make this review more sound and cohesive, we decided to categorize the biosensors into two major groups: **tools for direct measurement of cAMP** and **tools for indirect cAMP probing**. As the name implies, direct probes provide *first hand* cAMP measurements, with a readout typically being generated immediately after binding between cAMP and the sensor molecules. Additionally, the readout intensity from direct biosensors is usually proportional to the intensity of stimulation, which allows the direct probes to convey valid data on actual oscillations of intracellular cAMP levels. In contrast with direct probes, indirect biosensors typically measure the effects of cAMP on its downstream effectors. In case of indirect cAMP sensing, there is always at least one *intermediary* between actual concentration of cAMP and the readout from the probe. Obviously, in order to deduce genuine cAMP levels from such an indirect readout, one has to be aware of the relations between cAMP and its effector being probed and to account for possible interference that may happen on the way of signal relay. Though the above inherent factors limit the use of indirect sensors for measurement of actual levels of cAMP, the very nature of these sensors makes them excellent tools for studies of cAMP downstream effects.

In the following sections, we consider the modern cAMP biosensors with respect to the above categorization and discuss the principal groups of probes in more detail, highlighting their strengths and limitations. After that, we provide some guiding landmarks that would be of some assistance to the reader facing an uneasy task of selection of the most appropriate tool for cAMP probing for a particular experiment involving living cells. We finish the article with a short prospect on selected genetically-encoded tools for cAMP modulation in living systems. Combinatorial use of these cutting-edge instruments with biosensors for cAMP creates the most powerful research platform, which opens new avenues for fruitful studies of the life cycle and signaling properties of this cyclic nucleotide.

## Tools for Direct cAMP Measurement in Living Cells

Irrespectively of the nature of readout produced upon cAMP binding (e.g., fluorescence or luminescence), direct sensors detect cAMP molecules by means of native or modified cyclic nucleotide-binding domains (CNBDs) invariably present in its structure. All the CNBD exploited in modern sensors have been adopted from cAMP-binding domains of the three principal downstream cAMP effectors: PKA, exchange proteins activated by cAMP (Epac) or cyclic nucleotide-gated channel (CNGC; Kamenetsky et al., 2006; Willoughby and Cooper, 2007).

**FIGURE 1:**
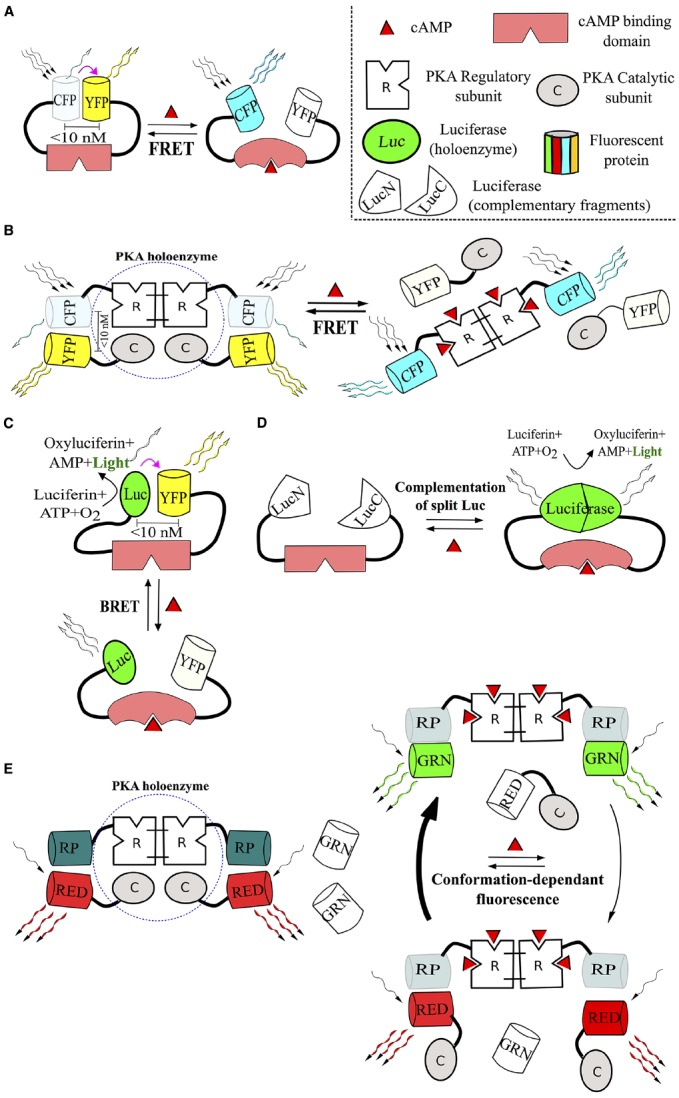
**Main classes of direct biosensors for cAMP.** A prototypical structure and mechanism of action of unimolecular and multimolecular FRET sensors (**A** and **B** respectively), BRET sensors **(C)**, sensors, based on luminescent enzymes **(D)**, and probes operating as conformation-sensitive fluorophores **(E)**, are depicted. All the listed sensors directly bind cAMP molecules and react to binding events with conformational changes that affect their signaling properties—FRET or BRET efficiency (purple arrow), intensity of light production or fluorescence (winding arrows). For more details, please, refer to corresponding sections of the text. Abbreviations: C, PKA catalytic subunit; CFP, cyan fluorescent protein; GRN, green fluorophore; Luc, luciferase; LucN/C, fragments, forming luciferase holoenzyme; R, PKA regulatory subunit; RED, red fluorophore; RP, regulatory protein; YFP, yellow fluorescent protein.

A prototypical direct biosensor for cAMP is a conformationally flexible multidomain protein, which undergoes a structural change once cAMP molecule lands in the pocket of the CNBD, followed by a change in the signaling intensity of the sensor, which is used to deduce the concentrations of cAMP the sensor is exposed to. Below we review the principal classes of direct cAMP sensors based on the type of signal they generate in response to cAMP (Figure 1).

### Förster Resonance Energy Transfer (FRET)-Based Sensors

Introduced in the early 1990s, biosensors based on FRET phenomenon have been instrumental in shaping of our understanding of the actual principles governing cAMP generation, flow, crosstalk and decay in defined compartments of living cells (Willoughby and Cooper, 2007; Lefkimmiatis and Zaccolo, 2014). Not surprisingly, FRET sensors remain the most popular and widely used tool for cAMP studies in living cells, with several dozens of distinct probes generated over the last three decades. For this reason, we will not discuss all the available cAMP FRET-based sensors in very detail—the reader is addressed to several comprehensive review papers instead (Willoughby and Cooper, 2008; Sprenger and Nikolaev, 2013; Gorshkov and Zhang, 2014). Instead, we shall try to highlight the basic principles of the method, advantages it offers and limitations one should keep in mind once planning an experiment with FRET-derived readout.

Förster resonance energy transfer refers to a mechanism of non-radiative energy transfer between a pair of light-sensitive molecules (fluorophores), a donor and an acceptor, that have partially overlapping spectral characteristics. For the transfer of energy, fluorophores making a FRET pair should be in close proximity (typically, within several nm from each other) and have a favorable relative orientation. As the efficiency of donor—to—acceptor energy *flow* is inversely proportional to the six power of the distance between fluorophores of any given FRET pair, the method is exceptionally sensitive to tiny alterations in FRET pair collocation (Forster, 1946; Correa and Schultz, 2009). This way, FRET intensity and its alterations provide an excellent reflection of a distance change between two fluorophores.

Förster resonance energy transfer intensity is typically measured by calculating a ratio of emission intensities of donor and acceptor molecules registered upon donor fluorophore excitation with light of the appropriate wavelength. Another way to estimate the degree of FRET is to photobleach an acceptor fluorophore and to track the rise in donor emission intensity. FRET efficiency estimates obtained with any of the above approaches are ratiometric in essence and are usually corrected for possible bleed-through of donor emission into the acceptor channel (Correa and Schultz, 2009).

In general, all FRET biosensors are designed to operate in the similar fashion. A prototypical FRET sensor represents a complex molecular structure with three principle domains: a sensor domain, that is in charge of binding to a molecule or compound of interest to be measured, and two fluorophore domains making a FRET pair. A binding of a target molecule to a sensor domain triggers a chain of conformational changes in a biosensor tertiary structure, which should lead to a change of distance between donor and acceptor fluorophores and result in alteration of FRET efficiency. Though multitude of variations in sensor design are possible, the above operational principle remains valid for all FRET biosensors, with the ones developed for cAMP not being an exception.

Since the number of molecules forming a sensor is an important factor that has several significant implications (reviewed below), we are considering all FRET cAMP indicators in the context of two big “*families*”: a family of **multimolecular sensors**, members of which are build up of at least two different molecular units carrying separate domains, and a family of **unimolecular sensors** having a sensor and a FRET pair within a single molecule. Units of a multimolecular sensor can either dimerise or dissociate, and any of these events should be accompanied by a measurable change in FRET intensity.

#### Multimolecular FRET Sensors for cAMP

In essence, all of the multimolecular FRET sensors for cAMP generated so far represent different genetically engineered modifications of PKA, one of the principal downstream effectors of cAMP. PKA holoenzyme is a heterotetrameric complex conformed by two subunits: the catalytic (C) and the regulatory (R) subunit, arranged in a C2:R2 ratio. Binding of cAMP to the R subunits results in conformational change and the dissociation of the two, now active, C subunits from a tandem of R units, that remain attached to each other.

More than 25 years ago, the group of Tsien reported on the first successful use of FRET sensor for cAMP measurement in living cells (Adams et al., 1991). This sensor, named FlCRhR, represented the chemically modified PKA, which had its C subunits labeled with fluorescein fluorophore and R subunits tagged with rhodamine (hence the name: Fluorescein + Catalytic unit/Rhodamine + Regulatory unit) in FRET distance in the inactive holoenzyme. Binding to cAMP evoked PKA holoenzyme dissociation with measurable drop in FRET efficiency, as fluorescein and rhodamine were not in the immediate vicinity any longer for successful FRET to occur.

FlCRhR turned out to be a groundbreaking tool, as it allowed for real-time measurement of intracellular cAMP in physiological concentrations and provided a wealth of information on spatial cAMP dynamics. However, this sensor was produced *ex vivo* by means of chemical labeling and hence had to be introduced into cytoplasm of single cells via microinjection that was obviously technically demanding and significantly limited the processivity of the method.

Such limitation was overcome a few years later with development of a set of genetically-encoded FlCRhR analogs, that could be expressed in living cells by means of transfection or viral transduction (Zaccolo et al., 2000). Additionally, PKA-based FRET sensors evolved improved characteristics in successive generations, including broader dynamic range and higher resistance to photobleaching (Lissandron et al., 2005).

Nonetheless, despite all the modifications, PKA-based sensors still bear several common shortcomings, and most of these are actually related to the very nature of PKA and its mode of operation. Firstly, despite being labeled with a pair of FRET fluorophores, PKA holoenzyme—based sensors preserve catalytic activity. This way, a rise in cAMP triggers the complex chain of signaling events, involving effectors downstream of PKA and altering transcriptional activity of cAMP-dependent genes (Beavo and Brunton, 2002; Cooper, 2003; see also the section of this review on Reporter Gene-Based Systems). These effects form a potential basis for short and long-term interference, which in some cases might not be immediately obvious and quite difficult to correct for, e.g., in prolonged studies with either chronic or multiple exposure to certain agents. Secondly, as PKA holoenzyme naturally dissociates upon cAMP binding, one should expect the reassembled holoenzyme to have incorporated endogenous non-fluorescent C subunits inevitably present in cell cytoplasm. Obviously, this might lead to loss of overall FRET efficiency with each dissociation–reassociation cycle, as the fraction of reassembled PKA holoenzymes will not carry FRET pairs any more. Besides, it should be recognized, that more complex structural dynamics of PKA-based probes, involving integration of a tetrad of subunits, might negatively affect temporal resolution of these sensors, potentially making them inferior to unimolecular probes in this regard (Rich et al., 2014).

In order to alleviate unwanted interference from endogenous PKA subunits, PKA-based sensors are to be heavily expressed. However, high levels of sensor expression combined with its naturally high affinity to cAMP might lead to significant cAMP buffering, thus distorting genuine patterns of cAMP spatio-temporal dynamics (Rich and Karpen, 2002). Another related issue is the necessity of equal expression levels of both PKA subunits, which are usually encoded by separate vectors and hence require co-transfection (Zaccolo et al., 2000). Lastly, some experimental evidence suggest that PKA might be activated by 3′, 5′-cyclic guanosine monophosphate (cGMP), which opens new avenues for interference and might lead to decrease in assay specificity (Forte et al., 1992).

The multimolecular structure and cooperative nature of action of PKA-based cAMP probes underpin significant part of the above limitations, which in essence are inherent to most multimolecular FRET sensors. Conversely, unimolecular FRET sensors, developed in the ensuing years, allowed to bypass some of the *old* problems and made a valuable addition to the cAMP toolbox (discussed below).

#### Unimolecular FRET Sensors for cAMP

The majority of unimolecular FRET sensors for cAMP currently available are based on genetically modified Epac proteins—exchange factors, directly activated by cAMP. Epac1 and Epac2 are closely related multidomain proteins that basically act as guanine nucleotide-exchange factors for the small GTPases Rap1 and Rap2. Binding of a single cAMP molecule to the CNBD of Epac1/2 triggers a conformational change with ensuing exposure of initially *shielded* catalytic domain, allowing it to bind and activate Rap1/2. In such fashion, Epac proteins, apart from PKA and CNGCs, are recognized as another immediate downstream effectors of cAMP (Bos, 2006).

The first reports on generation of Epac-based FRET sensors and their successful use for cAMP measurements in living cells trace back to 2004, when three independent research teams published their data (DiPilato et al., 2004; Nikolaev et al., 2004; Ponsioen et al., 2004). Since then the engineering activity in the field of unimolecular biosensors does not seem to cease and a host of improved sensor modifications have been developed, showing better signal-to-noise ration, enhanced temporal resolution and decreased sensitivity to light (Van der Krogt et al., 2008; Gorshkov and Zhang, 2014; Klarenbeek et al., 2015). All Epac-based sensors have a unimolecular structure and follow the similar design: a native or modified CNBD from Epac protein is typically decorated by two fluorophores involved in FRET. Binding of cAMP to the CNBD evokes a change in the sensor’s tertiary conformation, which is accompanied by alteration of FRET intensity.

Unimolecular FRET sensors provide several advantages over multimolecular cAMP probes. As all the functional domains are localized within a single molecule, the kinetics of conformational changes is more favorable—hence, unimolecular sensors have a faster response to cAMP (= higher temporal resolution) in comparison to more *bulky* multimolecular PKA-based sensors that require binding of four cAMP molecules (Nikolaev et al., 2004). Again, with unimolecular sensors only a single vector is needed for transfection and the unbalanced expression of different subunits that might be observed with multimolecular PKA sensors is no longer an issue. A handful of catalytically *dead* Epac sensors have been developed as well, thus eliminating the possible interference from Epac downstream targets (Nikolaev et al., 2004; Ponsioen et al., 2004; Klarenbeek et al., 2011). Endogenous Epac molecules have a lower sensitivity to cAMP than PKA holoenzyme (Bos, 2006). In line with this, PKA-based sensors are readily activated by nanomolar levels of cAMP, but affinities of Epac probes typically lie within low micromolar range (Bacskai et al., 1993; Ponsioen et al., 2004). Yet, higher sensitivity of PKA sensors comes with the price of early saturation and a narrowed dynamic range. Considering this, Epac-based probes appear advantageous, as they allow for measurements of cAMP at higher, but more physiologically relevant levels.

Apart from Epac probes, a few other unimolecular FRET sensors for cAMP have been engineered. One example is PKA-camps sensor, that represents a truncated regulatory IIβ-subunit (RIIβ) of PKA flanked by yellow and cyan fluorescent proteins (YFP and CFP respectively) making a FRET pair. PKA-camps has no catalytic activity and operates in the same fashion as Epac sensors: binding of one molecule of cAMP to RIIβ triggers a conformational rearrangement, manifested by FRET efficiency changes (Nikolaev et al., 2004). HCN2-camps is another unimolecular FRET probe for cAMP, which is based on a single cAMP binding domain of murine hyperpolarization-activated CNGC sandwiched between YFP and CFP. HCN2-camps has a lower sensitivity to cAMP and extended dynamic range (1–100 μM). Therefore, HCN2-camps is not prone to early saturation and thus *tailored* to cell types with elevated basal levels and amplitude oscillations of cAMP, e.g., cardiomyocytes (Nikolaev et al., 2006; see also the section of this review on CNGCs).

Though most of the FRET biosensors are designed for direct binding (and hence sensing) of cAMP, there are several related genetically-encoded FRET probes, that can be used for indirect measurement of cAMP levels, using PKA activity as readout. The so-called AKAR sensors (A-Kinase Activity Reporters) operate in the very similar fashion to unimolecular FRET sensors for cAMP and hence are briefly discussed herein. AKARs have a prototypical tetra-domain structure comprised of a PKA-specific substrate and a phosphoamino acid-binding unit sandwiched between two fluorophores making a FRET pair. Upon cAMP binding to PKA holoenzyme the released and activated C subunits phosphorylate the substrate sequence of AKARs, thus making it an appealing *bait* for the neighboring phosphoamino binding domain. This results in a change of sensor tertiary conformation, leading to alteration in FRET efficiency (Zhang et al., 2001; Allen and Zhang, 2006; Erard et al., 2013). It should be noted that AKARs have all the functional domains residing in a single protein, and as such enjoy all of the benefits of modern unimolecular sensors, including possibility of targeting to defined intracellular compartments (Allen and Zhang, 2006; Depry et al., 2011). On one hand, the indirect nature of the readout provided by AKARs and the propensity of PKA to amplify the incoming signal from cAMP, alongside with difficulties of calibration, render AKARs suboptimal tools for cAMP measurement, while on the other hand, when used for *direct indication*, i.e., for PKA activity studies, AKARs can show excellent performance.

Both multi- and unimolecular FRET sensors share a principle advantage—targetability to discrete subcellular compartments. By introducing mutations to native domains (e.g., alteration or removal of Disheveled/Egl/10-Pleckstrin domain of Epac, responsible for membrane anchoring) or fusing Epac proteins or PKA subunits with appropriate targeting signals, e.g., nuclear or mitochondrial localization signals, or membrane anchoring motifs (farnesylation, palmitoylation, or polybasic sequences), several sensors were successfully routed to desired compartments, including plasma membrane, cytoplasm, mitochondria, and nucleus (DiPilato et al., 2004; Ponsioen et al., 2004; Dyachok et al., 2006; Terrin et al., 2006; Sample et al., 2012). This very ability to provide outstanding spatial resolution, unattainable with other biosensors, combined with quick response and reversible nature of conformational changes induced upon cAMP binding, underpins the true power of FRET sensors as tools for cAMP measurement in living cells. Currently FRET sensors remain the tools of choice once cAMP real-time dynamics in defined subcellular regions comes to question.

All modern FRET sensors for cAMP are genetically-encoded proteins and hence can be used not only in cell cultures, but in laboratory animals as well, providing insights into cAMP life-cycle in non-perturbed microenvironment (Nikolaev et al., 2006; Calebiro et al., 2009; Jacobs et al., 2010; Sprenger et al., 2015). The ratiometric nature of FRET brings additional benefits, allowing to correct for cell-to-cell variation in sensor expression levels and to minimize the effects of auto-fluorescence and light scattering. Besides, FRET biosensors do not require additional expensive substrates for signal generation, as it is the case for all bioluminescence-based methods (see below).

Nevertheless, just like as all the other methods in life sciences, FRET sensors for cAMP have certain limitations. Firstly, FRET methodology is heavily dependent on microscopy and image analysis. Though a basic microscopy set-up suitable for FRET should be affordable for most labs (Börner et al., 2011), conversion of images into figures (i.e., from pixel intensity to cAMP levels) requires manual image processing, which is not only laborious, but might inadvertently lead to introduction of bias, e.g., while defining regions of interest to be analyzed (Rich et al., 2014). However, computer-assisted tools for automated image processing and emerging methods of signal acquisition and interpretation such as hyperspectral imaging already offering new solutions to old problems and hold promise for the future (Francis et al., 2012; Leavesley et al., 2013).

Another facet of complicated image processing is a relatively poor suitability of FRET-based biosensors for high-throughput applications. Though it is possible to run FRET assays in multiwell plate format on a fluorescence platereader and some attempts to increase throughput have been made (Allen and Zhang, 2006), currently other tools (see the section of this review on luciferase-based biosensors) appear to be preferential when high processivity or compound screen are desired.

When dealing with fluorescent proteins, it is prudent to account for possible effects of pH, temperature and other environmental factors, as these variables are known to influence performance of fluorophores and hence FRET efficiency (Correa and Schultz, 2009; Betolngar et al., 2015). This issue might become more pronounced, when targeted FRET biosensors are used, as sensor exposure to certain environmental factors might be different in distinct subcellular domains (e.g., pH and ROS levels in mitochondria; Putnam, 2012; Rich et al., 2014). Resistance to photobleaching is another variable affecting ultimate performance of a FRET pair to keep in mind once choosing the right sensor for experiment. At the same time, there has been a significant progress in the field, with recent generations of FRET sensors for cAMP appear to be more resistant to pH and photobleaching (Klarenbeek et al., 2011, 2015; Salonikidis et al., 2011).

It should be mentioned, that the conversion of FRET signal into absolute values of cAMP is not a trivial task. The most frequently used approach relies on calibration of a purified sensor against different concentrations of cAMP *in vitro*—however, this set-up is obviously unable to mimic physiologic conditions a sensor is exposed to inside living cells (Börner et al., 2011). An alternative and more physiological calibration method that has been recently introduced involves perfusion of sensor-carrying cells with different concentrations of cAMP via patch-clamp pipette, so the sensor remains and responds to cAMP in the native milieu (Koschinski and Zaccolo, 2015). Still, this protocol is obviously quite technically demanding and requires ample expertise with patch-clamp.

Last, but not least, FRET biosensors, like any other genetically encoded proteins, tend to have relatively high, non-physiological levels of expression, which may lead to excessive binding and thus *buffering* of cAMP. *Buffering* ability of a sensor becomes of more concern once targeted proteins are used, resulting in high local concentrations of cAMP-avid sensor molecules within certain subcellular compartments. The *buffering* phenomenon might distort natural patterns of cAMP synthesis, transport and breakdown, limiting a sensors ability to convey actual information on cAMP changes in living cells (Willoughby and Cooper, 2008). Noteworthy, cAMP *buffering* is not a unique limitation of FRET probes—this problem is inherent to all genetically encoded cAMP sensors that function as cAMP binders.

### Bioluminescence Resonance Energy Transfer (BRET)-Based Sensors

In essence, BRET and FRET are closely related phenomena, that are governed by the same physical laws and rely on the same principle of non-radiative energy transfer between a couple of closely opposed molecules (Forster, 1946). The only difference between FRET and BRET is in the type of a donor molecule: in case of FRET a fluorophore acts as a donor of energy, but in BRET a bioluminescent protein is used instead.

Enzymatically active variants of *Renilla reniformis* luciferase (Rluc) are typically used for donor molecules in the majority of BRET pairs. Oxidation of the substrate (coelenterazine) by Rluc results in the release of energy, which is either emitted as photons of light or transferred to an acceptor fluorophore, provided the latter is in the close proximity. In this fashion, no external source of light to excite donor molecule is required, which underpins most of the advantages of the BRET approach, including absence of photobleaching, phototoxicity and autofluorescence (Bacart et al., 2008). Besides, BRET is applicable to studies of light-dependent and light-sensitive processes, e.g., visual perception and photosynthesis (Saito et al., 2012). Lastly, such experimental tools as optogenetics (discussed below) and caged compounds liberated upon exposure to light can be combined with BRET with minimal chances of interference, which is not the case with FRET, requiring strong external illumination (Ellis-Davies, 2007; Fenno et al., 2011).

Only a handful of genetically encoded sensors suitable for live cell measurements of cAMP based on BRET phenomenon have been developed so far. All of these essentially exploit the same design principle and operate in similar fashion as the multi- and unimolecular FRET cAMP sensors reviewed earlier. The only available multimolecular BRET sensor for cAMP probing to our knowledge is based on PKA holoenzyme, which has its regulatory and catalytically subunits decorated with Rluc and a green fluorescent protein (GFP) variant, respectively. Hence, PKA holoenzyme dissociation upon cAMP binding is paralleled by pronounced drop in BRET, captured as a ratio of the emission values (Prinz et al., 2006). Unimolecular BRET cAMP indicators typically feature a cAMP binding motif of either Epac or PKA regulatory subunit sandwiched between a luciferase and a fluorescent protein, making a BRET pair (Jiang et al., 2007; Barak et al., 2008; Saito et al., 2012).

A slightly different design principle was exploited by Saito et al. (2012), who inserted Epac1-derived cAMP binding motif into the non-structural loop of a chimera YFP (Venus)-Rluc. The resulting sensor, named Nano-lantern (cAMP1.6), has the Rluc separated by the Epac1 motive into two complementary parts, rendering enzyme inactive as far as the cAMP-binding domain remains vacant. cAMP binding to the sensor evokes a conformational change and the restoration of Rluc structure and enzymatic activity, leading to BRET with the chimeric Venus protein. In such fashion, Nano-lantern (cAMP1.6) combines BRET and complementation of split luciferase principles, providing lower background signal and increased sensitivity for the assay.

Similar to FRET probes, BRET sensors are applicable to transgenic animals and can be targeted to desired subcellular compartments by means of introduction of appropriate localization signals (Barak et al., 2008; Saito et al., 2012). However, the intensity of BRET signal is still much weaker than emission from FRET indicators, which presently hinders efficient use of BRET sensors for single cell measurements or for tracing cAMP fluctuations in subcellular domains (Willoughby and Cooper, 2008; Saito et al., 2012).

In general, BRET sensors have a favorable dynamics of spatial rearrangements (several seconds) in response to changes in cAMP levels. The fast and reversible nature of BRET response makes the indicators of this class well-suited for real-time measurement of cAMP in pools of living cells (Jiang et al., 2007). Another principal advantage of BRET sensors is their applicability for high-throughput screening (HTS). As generation of BRET signal is not dependent on external source of light, the signal capture becomes much easier. BRET sensors do not need dedicated microscopy set-up, required for FRET sensors, and the assays can be easily carried out in multiwell plates on a platereader equipped with the appropriate filter set. Simplified readout expressed as a ratio of two emissions obviates the tedious image processing typical of FRET and adds to the processivity of the assay (Boute et al., 2002; Jiang et al., 2007; Bacart et al., 2008).

At the same time, apart from the highlighted specialties, BRET and FRET-based cAMP indicators are largely similar. Hence, most of the strong points and shortcomings inherent to unimolecular and multimolecular FRET sensors reviewed in the previous section remain valid for BRET sensors of similar design as well.

### Direct Sensors for cAMP, Based on Luminescent Enzymes

Though luminescent enzymes have an impressive record as reporters in functional studies of gene expression, their use as biosensors for direct probing of various intracellular molecules has started less than a decade ago (Hill et al., 2001; Jiang et al., 2008; Binkowski et al., 2009).

At large, all sensors of this class are genetically encoded proteins of similar operational structure, which are based on a variant of luciferase fused with functional domains responsible for sensing of desired analytes. Interaction of a sensing domain with its ligand typically triggers conformational rearrangement of the luciferase, altering its enzymatic activity and changing the output of light.

Bioluminescent sensors only require appropriate substrate (e.g., luciferin or coelenterazine) and molecular oxygen for generation of photons and hence are not dependent on exogenous light source. As most of biological samples do not produce any light *per se*, this feature of luminescent probes not only endows them with excellent sensitivity and signal-to-noise ratio, but also helps to avoid a multitude of problems inherent to fluorescent proteins and fluorophore-based sensors (discussed in the previous section).

A few dedicated biosensors for cAMP based on mutated variants of luminescent enzymes have been engineered so far. An elegant approach is the dual-wavelength cAMP indicator engineered by Takeuchi et al. (2010). This unimolecular genetically-encoded sensor is based on *N*-terminal fragments of two different click beetle luciferases (ElucN and CBRN) and one *C*-terminal fragment (McLuc1) which could dimerize with any the above *N*-terminal fragments, forming two distinct luminescent enzymes with well-separated emission peaks (613 nm for ElucN and 538 nm for CBRN).

The cAMP binding domain of PKA RIIβ, that is linked to the luciferase fragments by peptide bridges, serves for cAMP sensing unit. In the absence of cAMP, McLuc1 and ElucN form a functional enzyme that generates red light. Upon cAMP binding to PKA RIIβ the sensor undergoes conformational rearrangement, leading to separation of the red light-producing luciferase and *migration* of McLuc1 toward CBRN, culminating in restoration of CBRN holoenzyme and green light production. This way, the sensor was originally designed to provide ratiometric readout of cAMP levels expressed as a shift of red to green light emission intensities.

Sadly, the resulting sensor refused to *behave* strictly according to the initial design assumptions: although cAMP binding did result in generation of green light, this was not paralleled by a drop in red light emission. This irresponsiveness of the red light luciferase to probe occupancy with cAMP narrows the signal-to-noise ratio of the sensor and deprives it of all the benefits that genuine ratiometric readout provides, e.g., insensitivity to differences in sensor expression levels or variations in substrate availability. Nevertheless, apart from this issue, the indicator has a good cAMP sensitivity range (1.0 × 10^–7^ to 1.0 × 10^–4^ M) and is easy to use for semi-quantitative cAMP probing in cell populations, including high-throughput setting. Though the relatively weak light intensity precludes studies of cAMP in subcellular compartments, the sensor can produce fair kinetic data on total cAMP oscillations in cellular pools over extended periods of time.

Another example of cAMP luminescent indicators is a dyad of similar sensors (pGloSensor-20F and its successor with improved characteristics pGloSensor-22F), based on a on circularly permutated variant of a firefly luciferase fused with cAMP-binding domain B of PKA RIIβB, which were developed by Wood and colleagues (Fan et al., 2008; Binkowski et al., 2011a). In these probes, RIIβB domain acts as an allosteric regulator of the adjoined luciferase—“*landing*” of a single cAMP molecule in the RIIβB pocket allows the silent luciferase to regain the enzymatically active conformation, which in the presence of the substrate (luciferin) immediately leads to light production. With a detection limit in low nanomolar range and outstanding dynamic window (0.003—100 μM for the improved 22F version of the sensor), GloSensor proteins are some of the most sensitive cAMP sensors presently available. These features, combined with the benefits of using the light emission as a readout (very low levels of non-specific signal) and broad linearity of response to cAMP, endow the probes with unsurpassed signal-to-noise ratio of up to 800-fold (Binkowski et al., 2011a). On top of it, fast dynamics and reversibility of sensor conformational changes in response to alterations in cAMP levels (within several seconds) make GloSensor probes excellent tools for direct tracing of real-time cAMP dynamics in living systems.

Encoded by a single plasmid, GloSensor probes avoid difficulties related to non-equality of expression of different domains making a functional sensor, that are inherent to, e.g., CNGCs and multimolecular FRET probes, and can be used in a variety of cell types and in principle should be applicable to laboratory animals. Importantly, due to simplicity of signal capture, attainable by a plain luminometer or luminescent platereader, and ease of data analysis, the probes are quite user-friendly. Most of the above features render GloSensor proteins readily suitable for high-throughput tasks, e.g., massive library screens of putative GPCRs interacting compounds (Pantel et al., 2011).

At the same time, the luminescent signal from GloSensor probes is not easily convertible to absolute cAMP values. In principle, the sensors can be calibrated against any end-point assay measuring cAMP levels in cell lysates. Calibration of this kind, however, is not quite straightforward and reliable, as the intensity of luminescence in living cells transfected with GloSensor probes appears to be affected by several factors, including temperature, availability of the substrate and sensor protein expression levels, which might not be easy to account for (Binkowski et al., 2011b). Due to limited spatial resolution GloSensor probes are mainly suited for measurements of cAMP in cell populations rather than in single cells. Though GloSensor proteins technically may be targetable to certain subcellular compartments by fusion with localization signals, no reports on generation of that kind of modified sensors have been published so far. Altogether, when studies of intracellular cAMP spatial organization are in question, biosensors with higher spatial resolution, e.g., FRET-based probes, offer a better solution.

Of note, GloSensor proteins have been harnessed for indirect measurements of cAMP in non-transfected primary cell cultures by means of co-incubation of primary cells (donor cells) with a cell line stably transfected with GloSensor probe (*sensor* cell line), in an assay called CANDLES (cyclic AMP iNdirect Detection by Light Emission from Sensor cells; Trehan et al., 2014). This coculture set-up allowed for bi-directional transport of cAMP molecules via gap junction channels formed by neighboring cells. In such fashion, a rise in cAMP concentration in donor cells, triggered upon activation of GPCRs expressed exclusively in these cells and not in the *sensor* cells, could be registered in a kinetic fashion by a luminescent signal from the *sensor* cells.

Although the authors showed the interspecies detection of cAMP by this method, this assay might not be applicable to all cell types, even from the same organism, as some cells might be *reluctant* to form gap junctions with *sensor* cells, thus rendering intercellular flow of cAMP impossible. Another drawback of this method lies in its dependence on PDE inhibitors [such as 3-isobutyl-1-methylxanthine (IBMX)] to inhibit cAMP degradation (Trehan et al., 2014). Inability to register significant flow of cAMP molecules between the cells once cAMP breakdown machinery was fully active and not silenced with IBMX limits the *resolution* of the method, as the readout captured in the absence of PDE activity reflects severely distorted patterns of cAMP kinetics and intracellular distribution.

Apart from this, it should be recognized, that the above coculture set-up allowing for cAMP studies in primary cultures in principle might be implemented with any other modern indicator for cAMP expressed in *sensor cells,* e.g., with Epac and PKA-based FRET probes, as was successfully demonstrated by Ponsioen et al. (2007).

Lastly, when planning experiments with luminescent-based enzymes, do not forget to account for one additional universal drawback of this methodology—its dependence on a substrate for the luciferase. This not only increases the expenditure on the assay, but may also affect the resulting readout, as the concentration and availability of the substrate are important factors for signal generation. Moreover, some substrates are short living, affected by pH and temperature, and hence limiting the assay’s capabilities and usability.

### cAMP Sensors, Based on Conformation-Sensitive Fluorophores

The story of conformation-sensitive fluorophores starts from the pioneering work of Tsien and colleagues, that culminated in development of a novel class if Ca^2+^-biosensors, coined “*camgaroos*” (Baird et al., 1999). Subsequently, the main principle exploited in *camgaroos* was tailored to other applications, providing the research community with a handful of robust genetically-encoded indicators for various intracellular targets in living cells, including Ca^2+^, cGMP, and some kinases (Whitaker, 2012; Gorshkov and Zhang, 2014).

In essence, the indicators from this family rely on different circularly permutated variants of enhanced GFP, which fluorescent properties are conformation-dependent. A prototypical conformation-sensitive indicator consists of two principal domains, a sensor and a fluorescent reporter, that are fused together. A sensor domain is designed to bind a desired target [e.g., calmodulin (CaM) for Ca^2+^ cations], and the binding event is ultimately relayed into a conformational change of a reporter protein, leading to a change in its fluorescence. Though the readout is typically expressed as a ratio of two emissions intensities (i.e., registered in basal and stimulated states of a sensor), its fundamentally different from FRET, as all the fluorescence is coming from a single fluorophore.

Despite the appealing design principle, no conformation-sensitive non-FRET sensors for direct probing of cAMP have been developed so far. Bonnot et al. (2014) reported the generation of single fluorophore sensor for PKA activity in living cells, that represents a modification of the original AKAR2 FRET biosensor (see also the section of this review on FRET-based probes). Conformation-sensitive mutant of GFP in this probe responds to phosphorylation of the attached PKA substrate by a change in fluorescence intensity and lifetime. Though this genetically encoded indicator may be used for indirect probing of cAMP levels, it is obviously inferior to direct sensors for cAMP and should be reserved for the original application—studies of PKA activity.

Another related methodology based on conformation sensitive fluorophores that is applicable for cAMP measurements in living systems have been recently published (Ding et al., 2015). The assay general principle is based on heterologous co-expression of a trio of interacting proteins in a single cell. While two of the proteins carry fluorophores with different excitation/emission spectra, the third member of the trio lacks any fluorescent properties, but is able to dimerize with either fluorophores, acting as their allosteric regulator. In monomeric (or unbound) state, the fluorescent proteins are quenched and unresponsive to light stimulation, but dimerization with the allosteric regulator triggers conformational rearrangement of the pair, leading to dramatic rise in fluorescence. Provided that the rate and preferential direction of dimerization events between the regulator and the fluorophore-carrying proteins are governed by defined stimuli (e.g., intracellular calcium levels or certain enzymatic activity), that can be achieved by fusion monomeric proteins with various functional domains or *bridging* them with recognition sequences for desired enzymes, this trio of proteins make an extremely versatile platform, that have already been successfully used for probing of cAMP, calcium–calmodulin interactions, caspase-3 and extracellular signal-regulated kinase activity (Ding et al., 2015).

For intracellular cAMP measurements in living cells, one of the fluorophores (red) and the regulatory protein were fused with the C subunit and R subunit of PKA, respectively, while the second fluorescent protein (green) was designed to remain in the cytoplasm in unbound state. This way, upon basal cAMP levels the red fluorophore and the regulatory subunit remain in close proximity, leading to strong emission of red light against the faint emission of green light from the quenched unbound second fluorophore. A rise in intracellular cAMP leads to PKA holoenzyme dissociation and liberation of the regulatory protein-R subunit chimera into cytoplasm, allowing it to bind the second fluorophore with resulting increase in the green-to-red emission ratio. As both fluorophores respond to cAMP in antipodal directions, it improves sensitivity of the assay, allowing for *recognition* of low-amplitude changes in cAMP concentration. However, due to the necessity of cotransfection with a set of vectors and inevitable differences in expression levels of the regulator and the fluorescent proteins, the assay provides qualitative rather than quantitative readouts. Measurements of absolute values of cAMP are further hindered by different binding affinities of the regulatory protein for its fluorescent counterparts.

To summarize, genetically encoded sensors for cAMP based on conformation-sensitive fluorophores make a new class of tools, which holds good promise for the future. These sensors are principally targetable to discrete subcellular compartments by fusion with relevant localization signals and thus might be used to gain insightful data on spatial organization of cAMP signaling. Simplified readout and data analysis (in comparison to FRET) makes conformation-sensitive probes more *user-friendly* and brings them closer to HTS. However, issues with calibration, absence of comprehensive data on kinetics of conformational alterations, narrow signal-to-noise ratio and vulnerability to pH changes leave some space for improvement and we are to await till this sensor class has found its niche in cAMP research field.

## Tools for Indirect cAMP Measurement in Living Systems

As was previously mentioned, indirect probes are originally designed to measure cAMP downstream effects rather than its absolute concentrations. Therefore, though one can get a good idea of cAMP levels with selected indirect probes, these tools are generally inferior to direct sensors in this regard. But if used wisely, indirect probes can provide a wealth of information on cAMP signaling outcomes and hence remain actively involved in cyclic nucleotide studies (Hill et al., 2010).

### Probes, Based on CNGCs

Cyclic nucleotide-gated channels comprise a big family of structurally related membranous ion channels, which are activated by cGMP and cAMP. Upon binding with the above cognate ligands, CNGCs undergo conformational change and become permeable for Na^+^, K^+^, and Ca^2+^, allowing these cations to enter cytoplasm, which ultimately leads to either a depolarization or a hyperpolarization event. In such fashion, CNGCs basically function as switches that sense changes in intracellular cyclic nucleotide levels and transform them into a change of membrane potential and Ca^2+^ concentration. Originally discovered in retinal photoreceptor and olfactory neurons, CNGCs have a pivotal role in signal transduction of vision and olfaction. Besides this, CNGCs have been shown to be of importance in functional regulation of several other cell types and organs, including kidney, sperm cells, endocrine tissues and pacemaker cells of the heart (Kaupp and Seifert, 2002; Craven and Zagotta, 2006; Biel and Michalakis, 2009).

Though CNGCs and related hyperpolarization-activated cyclic nucleotide-gated channels (HCNs) share the common CNBD and bear general structure similarity, they operate in somewhat different mode: CNGCs are solely cyclic nucleotide-dependent and become permeable for Na^+^, K^+^, and Ca^2+^ upon activation, while HCNs are mainly governed by voltage with cGMP and cAMP acting only as *fine regulators* that adjust activation threshold (activation voltage and activation time). In active state, HCNs preferentially allow entry of Na^+^ and K^+^ but provide very limited *access* to calcium ions (Kaupp and Seifert, 2002; Biel and Michalakis, 2009; Akimoto et al., 2014). Of importance, prolonged exposure to cyclic nucleotides does not lead to CNGCs desensitization (Dhallan et al., 1990).

In such a way, a change in transmembrane electric current and/or intracellular Ca^2+^ levels in CNGCs harboring cells in principle may be used as an indirect measure of cAMP oscillations. However, naturally occurring CNGCs are poor sensors for cAMP due to several reasons: The major *limitation* of wild-type CNGCs lies in their low responsiveness to cAMP and preferential avidity for cGMP (Rich et al., 2001; Kaupp and Seifert, 2002). Secondly, CNGCs activation threshold and sensitivity to cyclic nucleotides are under negative feedback control of calcium–calmodulin complex (Ca^2+^-CaM), which not only narrows the dynamic range of wild-type CNGCs-based assays, but brings possibility of interference with other signaling events, that affect intracellular Ca^2+^ levels (Liu et al., 1994; Trudeau and Zagotta, 2003). Another possible limitation to keep in mind is that wild-type CNGCs may be directly activated by nitric oxide (NO; Broillet, 2000).

In order to make CNGCs usable as cAMP sensors, a set of genetically modified CNGC subunits has been generated and tested in heterologous expression systems. Several combinations of different mutant subunits were shown to form functional CNGCs with desired properties, including higher affinity to cAMP and decreased cGMP responsiveness, loss of negative regulation by Ca^2+^-CaM due to alteration of CaM binding site and inability to respond to NO. These genetically engineered variants of CNGCs made the platform for cAMP measurements in a majority of successive studies (Altenhofen et al., 1991; Liu et al., 1994; Varnum et al., 1995; Rich et al., 2001; Reinscheid et al., 2003; Wunder et al., 2008).

Intracellular cyclic nucleotide levels in living cells were measured with CNGCs for the very first time by Trivedi and Kramer (1998), who used a change in membrane potential registered by a keen technique named *patch-cramming* for real-time probing of cytoplasmic cGMP levels in single living cells. Patch cramming involves heterologous expression of appropriate sensor channels in donor cells (chimeric CNGCs consisting of α—subunits from bovine rod and rat olfactory receptors were expressed in *Xenopus* oocytes in the above study), removal of a piece of cell membrane carrying CNGC and its calibration with different concentrations of cGMP via inside-out excised patch, with subsequent insertion (*cramming*) of the calibrated membrane into a recipient cell so the inside part of the sensor membrane is in direct contact with the cytoplasm of a recipient cell. This way, the sensor membrane on a tip of a pipette responds to changes in cytoplasmic cGMP levels of recipient cell by activation of CNGCs, leading to alterations in electric current that can be readily registered with patch clamp. The “patch cramming” approach allowed Trivedi and Kramer (1998) to follow cGMP levels in living neuroblastoma cells and rat neurons in real-time fashion, providing kinetic readout of cGMP oscillations scarcely attainable before. However, this methodology was obviously quite labor-intensive, involved multiple technically demanding steps and was applicable only to relatively large cells with a diameter of about 40 μm and higher.

A refinement followed a few years later—by means of viral transduction heterologous CNGCs and HCNs were directly expressed in the cells of interest and cytoplasmic cAMP fluctuations upon exposure to different stimuli were registered in single living cells in real time by means of whole-cell, inside-out, and perforated patch clamp (Rich et al., 2000, 2001; Heine et al., 2002). Thus, the limitations of the cell size and the need of harvesting a piece of CNGCs-carrying sensor membrane from donor cells were eliminated, facilitating more rapid and flexible assay flow.

Despite the above advances, CNGC-mediated measurements of cAMP were still invariably based on registration of electric currents by patch-clamp, which restricted the processivity of the method (one measurement—one cell) and required sophisticated probing tools and a skillful operator. The above limitations have been partially circumvented in the recent decade via development of automated patch-clamp instruments, though these tools are not readily available to majority of laboratories and remain to be tested for most of the cell types (Farre and Fertig, 2012).

Another general shortcoming of patch-clamp technique (excluding whole-cell setup) is that it can probe electric currents only in the immediate vicinity of a patch pipette tip, which restricts the measurements of cAMP to cell compartments that can be patched. For CNGCs, which expression is confined to plasma membrane, it means that patch-clamp allows to probe cAMP changes occurring exclusively in submembranous regions, providing little information on the events in other cellular compartments (Rich et al., 2000, 2014).

The aforementioned drawbacks can be bypassed by harnessing Ca^2+^ cytoplasmic oscillations as a measure of cAMP-driven CNGCs activation. Various methods for Ca^2+^ probing have been successfully applied to this aim, but most frequently Ca^2+^-sensitive cell permeant dyes such as fura-2/AM, indo-1/AM and fluo-3 AM/fluo-4 AM have been used (Grynkiewicz et al., 1985; Fagan et al., 2001; Rich et al., 2001; Visegrády et al., 2007). These established Ca^2+^ indicators provide either direct (increase in fluorescence quantum yield upon Ca^2+^ binding—fluo-3 and -4 dyes) or ratiometric (fura-2 and indo-1 dyes) measure of intracellular Ca^2+^ levels. Though both types of dyes are quite sensitive and easy to use, ratiometric indicators appear to be advantageous, as readout expressed as an emission/excitation wavelength shift allows to avoid unwanted effects of uneven loading, photobleaching and noise from non-specific autofluorescence, thus yielding higher signal-to-noise ratio and less variability.

Besides imaging of CNGCs-mediated Ca^2+^ surges in cytoplasm of a single cell with standard fluorescent microscopy, fluorescent Ca^2+^ dyes are readily applicable for measurements in cell populations, i.e., with the help of a multiwell platereader/spectrofluorometer equipped with appropriate filter sets, thus rendering this assay principle readily suitable for HTS (Reinscheid et al., 2003; Visegrády et al., 2007).

Apart from Ca^2+^-sensitive dyes, another approach initially developed for Ca^2+^ probing in living cells have been subsequently harnessed for cAMP measurements. Oscillations of intracellular Ca^2+^ triggered by cAMP-dependent activation of CNGCs can be registered with aequorin-based sensors (Sheu et al., 1993; Wunder et al., 2008; Richter et al., 2015). Consisting of two subunits, the apoprotein apoaequorin and its cofactor coelenterazine, aequorin is a well-studied bioluminescent protein from the jellyfish *Aequorea victoria.* Binding of free Ca^2+^ Ca^2+^ to aequorin triggers oxidation of coelenteramide, leading to generation of light and the holoprotein complex dissociation. The amount of photons emitted is proportional to Ca^2+^ ions concentration and can be easily registered with either a charge-coupled device (CCD) camera or a luminometer (Shimomura, 1985). In such fashion, successful co-expression of CNGCs and apoaequorin allows to measure cAMP in virtually any cell type. Besides, this methodology is not technically demanding and HTS-friendly (Wunder et al., 2008).

However, the generation of cell lines stably transfected with CNGCs and apoaequorin is quite time-consuming and might happen to be problematic, especially if one does not have all the components making the functional system in a single vector. Another limitation of this type of assay is the need of spiking the cells with Ca^2+^ in order to trigger aequorin break-up and light emission. This, together with short-lasting nature of Ca^2++^ responses ($\underset{˜}{<}$30 s), limits the processivity of the method. At the same time, the latter issue might be bypassed by running the assay in a multiwell format (e.g., 96- or 384-well plates) on a platereader that is equipped with liquid dispensing system.

Apart from the Ca^2+^-sensitive fluorescent dyes and apoaequorin-based assays, past decades witnessed the generation of another type of Ca^2+^ sensors, that have literally changed the field of calcium signaling research—this entails the class of genetically-engineered fluorescent proteins, capable of measuring Ca^2+^ oscillations by means of change in FRET intensity (Miyawaki et al., 1999; Whitaker, 2012). Though FRET sensors can provide excellent spatial resolution down to subcellular domains (discussed earlier) and obviously have a great potential for studies of crosstalk between cAMP and Ca^2+^ signaling, they have not been widely used in conjunction with CNGC.

Yet another quite similar approach for indirect intracellular cAMP measurement in living cells is based on membrane potential sensitive dyes such as DiSBAC_2_(3) and HLB 021-152. These molecules are impermeable to cells until plasma membrane depolarization occurs; once in the cytoplasm they exhibit a dramatic rise in fluorescence after binding with intracellular solutes and proteins, which can be registered with a conventional spectrofluorimeter or fluorescent microscopy. This assay format has been optimized for HTS as well (Tang et al., 2006; Visegrády et al., 2007).

**FIGURE 2:**
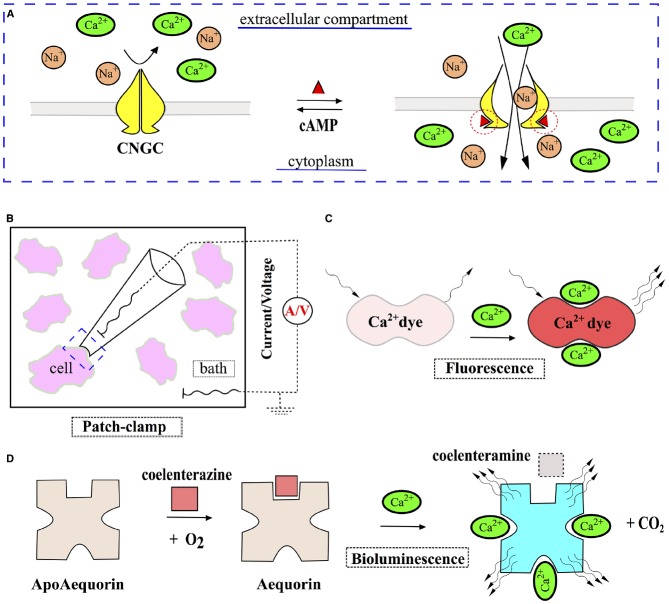
**Harnessing CNGCc for indirect measurement of cAMP. (A)** Basic operational principle of CNGCs: binding of cAMP (red triangle) to CNGCs results in channel opening for cations, allowing Na^+^ and Ca^2+^ to enter the cytoplasm. **(B)** Changes of the membrane potential and transmembrane electric currents, mediated by cAMP-driven CNGCs activation, can be registered with patch-clamp technique and serve as an indirect measure of cAMP levels. **(C,D)** Instruments, initially designed to measure concentration of intracellular Ca^2+^, can be applied in combination with CNGCs for indirect sensing of cAMP. Inorganic dyes (e.g., fluo-3), that demonstrate a pronounced rise in fluorescence upon binding of Ca^2+^ **(C)**, and bioluminescent protein apoaequorin, responding to Ca^2+^ by generation of light **(D)**, have been successfully used to this end.

To conclude, genetically-engineered CNGCs with increased sensitivity to cAMP represent a versatile analytical tool that can be tailored to wide array of research needs (Figure 2). Measurements of inward currents and membrane potential with patch-clamp allow one to have all the advantages that the very nature of CNGCs provides, including high spatial resolution, *snap-shot* responses to change in cyclic nucleotide levels within dozens of milliseconds, lack of saturation, excellent sensitivity and fair dynamic range, possibility to obtain absolute values of cAMP levels by means of excised patch calibration. However, these *benefits* are only available to dedicated and well-equipped labs with a strong record in electrophysiology and typically come at the expense of low processivity.

Ca^2+^-sensitive dyes and apoaequorin-based sensors are easier to use and suitable for HTS, although absolute levels of intracellular cAMP usually remain elusive due to infeasibility of calibration. Another shortcoming of using Ca^2+^ levels as a measure of cAMP is the possible interference from other pathways involving Ca^2+^ signaling.

Necessity of CNGCs expression in heterologous systems by means of transfection/transduction makes an inevitable common limitation of CNGCs-based assays. This issue becomes especially important once the different subunits comprising a functional CNGC are delivered by separate vectors or another exogenous genetically encoded sensor is used in conjunction (e.g., apoaequorin). Another thing to keep in mind is that high spatial resolution of cAMP measurements in submembranous regions achievable with CNGCs-based methods turns into a disadvantage once cAMP dynamics in another cellular domains comes into question, as membrane-anchored CNGCs can respond to cAMP only in the immediate vicinity of thereof. Last, but not least, despite rapid activation and kinetic-like pattern of signal, with CNGCs one should not expect to obtain genuine kinetic data on cAMP fluctuations over time, as the measurable response to cyclic nucleotides is indirect in essence and mediated by either inward ionic currents or Ca^2+^ levels. In inherently complex living systems there is virtually no place for strictly linear relations between any two given cross-talking pathways—and each of these has its own mode of signal transduction and tends to be intertangled with a number of *third parties*. Ca^2+^ signaling and transmembrane electric currents do not make an exception from the above.

### Reporter Gene-Based Systems

Reporter gene-based systems perhaps make the most illustrative example of indirect assay for cAMP, as these tools score on one of the most remote effects of cAMP, i.e., its effect on transcriptional activity. A rise in intracellular cAMP triggered by a variety of stimuli (most frequently, due to activation of ACs via Gα_*s*_—coupled GPCRs) leads to dissociation of PKA with successive entry of liberated C subunits into cell nucleus and the phosphorylation of cAMP-response element (CRE)-binding proteins (CREBs). Phosphorylated CREBs then bind to CRE elements within promoter regions of a multitude of genes, thus altering their transcription (Neves et al., 2002; Wettschureck and Offermanns, 2005; Musnier et al., 2010). In such way, a change in protein levels may be used as indirect measure of cAMP oscillations.

CRE consensus sequence (TGACGTCA) and its variations might be found in promoter regions of thousands of genes across human genome (Lalli and Sassone-Corsi, 1994; Zhang et al., 2005). However endogenous promoters have not been frequently used for cAMP studies, as they tend to encompass binding sites for other transcriptional factors as well, thus rendering reporter protein synthesis not solely cAMP-dependent (Hill et al., 2001). Genetically-engineered promoters harboring only CRE elements do not have this limitation and thus provide a viable alternative. Along with this, synthetic promoters are not totally problem-free and require thorough validation in order to yield the best transcriptional response, including testing of different CRE elements and various numbers of repetitive CRE sequences, as well as the reporter gene (Dinger and Beck-Sickinger, 2002; Shan and Storm, 2010; Hald et al., 2015).

The choice of reporter proteins is broad and the selected reporter generally should be tailored to particular needs. The possible options include different enzymes allowing for absorbance (e.g., alkaline phosphatase and β-galactosidase) or luminescence-based readout (different types of luciferases), fluorescent proteins and combinations of thereof (Hill et al., 2001; Jiang et al., 2008). If the assay in question is well-optimized and sensitive enough, all of the above reporters are able to provide a reasonable estimate of cAMP effects in living cells.

Despite relative ease of use, all reporter gene-based assays share a number of limitations, which should be taken into account already at the stage of experimental design. As all the reporter-based assays are dwelling on the readout coming from a protein product of a synthetic construct, this construct has to be delivered to cell nucleus for successful expression by means of transfection or viral-mediated transduction. Obviously, this brings along all the possible issues with transfection/transduction, including low success rates in certain cells types, significant inter-run variation in terms of transfection efficacy, excessive toxicity due to gene delivery procedure *per se* and poor applicability of the method to *in vivo* settings (Kim and Eberwine, 2010). However, as already discussed earlier, similar problems are in essence inherent to all of the genetically-encoded proteins and hence to the bulk of cAMP biosensors presently available.

Some of the transfection-related difficulties like run-to-run variation in reporter expression levels, leading to high heterogeneity of response and/or poor signal-to-noise ratios, may be circumvented by generation of stable cell lines with relatively uniform and equal expression of reporter. Of note, this strategy, although offering a good solution, is not flawless, as even with stable clones, the number of transgene copies, the location of the genomic loci of insertion and the state of the cells, e.g., passage number, might introduce massive variability (Jonas et al., 2013). The establishment of a stable line is a laborious process and does imply extra requirements to reporter construct, as it should confer antibiotic resistance for efficient selection of stable clones. Besides, the reporter construct should ideally encode a fluorescent tag allowing for negative selection of a fraction of stable clones with highest basal levels of reporter expression, providing the resulting cellular system with optimal sensitivity to cAMP (Hald et al., 2015). On top of it, the above approach is essentially non-applicable to primary cell cultures with a finite life span.

Another potential problem with reporter gene-based systems is related to basal promoter activity in the absence of its cognate transcription factor (CREB). This might happen when the strong promoter is being used (e.g., cytomegalovirus or phosphoglycerate kinase), thus leading to reporter protein synthesis in response to basal cAMP levels, which undermines assay performance and might lead to false-positives (Scarff et al., 2003; Hald et al., 2015). At the same time, this problem might be minimized via promoter *rejigging* or via prior negative selection of the cells exhibiting significant reporter signal in unstimulated state, thus yielding a homogeneous basally *silent* population. The latter strategy is feasible in cases when the reporter gene encodes for either a fluorescent protein (Kotarsky et al., 2001; Hald et al., 2015) or certain enzymes coupled to fluorescent readout (e.g., cleavage of FRET substrate CCF2/AM by reporter β-lactamase, leading to drop in FRET efficiency, as reported by Zlokarnik et al., 1998), thus allowing for facile removal of basally *active* cells via fluorescent-activated cell sorting.

*Excessive* reporter protein stability presents yet another possible limitation of reporter gene-based assays. Obviously, if a reporter protein is quite stable and has a natural decay period of more than several hours [which is the case with many conventional fluorescent or enzymatic reporters (Van Diggelen et al., 1980; Corish and Tyler-Smith, 1999)], this will have a huge impact on an assay sensitivity and dynamic range, as first reporter molecules that have been synthesized upon initial cAMP stimulation will still be present and actively *signaling* after several hours, thus adding to any resulting readout from successive surges of cAMP (Hill et al., 2001).

The above limitation might be partially circumvented by using genetically-engineered reporters destined for fast proteolytic degradation, as was successfully achieved by fusing PEST domain of mouse ornithine decarboxylase or mouse cyclin B1 destruction box to *C*- or *N*-termini of GFP, respectively, shortening GFP half-life to 2–6 h (Li et al., 1998; Corish and Tyler-Smith, 1999). Leclerc et al. (2000) applied similar strategy to a firefly luciferase, engineering the mutant enzyme with a functional half-life of only 50 min (in contrast to 4 h of wild-type enzyme).

Another way to obtain data on cAMP-driven kinetics of gene expression is to use a reporter that is released into cell medium, e.g., secreted isoforms of placental alkaline-phosphatase or β-lactamase (Cullen, 2000; Qureshi, 2007). By repeatedly removing cell medium and analyzing multiple sequential samples, one can minimize the effect of reporter protein accumulation and increase the dynamic range of the assay. Although this multiple sampling approach can convey some idea of intracellular cAMP fluctuations and expression of cAMP-dependent genes over time, it is still a way from deducing the genuine kinetics of cAMP-driven transcription, which is attributed to inherent features of signal transduction from the level of cAMP to CRE regions. As was previously shown by Hagiwara et al. (1993) slow entry pace of PKA catalytic units into cell nucleus, their low affinity for CREB and low concentrations of the latter basically promote a slow and relatively long-lasting transcriptional response with early saturation of the downstream signal, which is in striking contrast with seconds-lasting amplitude surges of cAMP within cytoplasm.

The amplification of the downstream signals on the way from the plasma membrane to the nucleus is a well-known phenomenon (Wettschureck and Offermanns, 2005). Considering this, the initial signal, encoded by a rise in cAMP upon GPCRs activation, will be augmented manifoldly by the time it reaches CRE loci of reporter genes in the nucleus. This general nature of signal transduction might be advantageous if one is studying the effects of low-amplitude oscillations of cAMP. However, in the presence of pronounced changes of cAMP the above benefit turns into a limitation, as the signal relay quickly gets saturated of on the levels of nucleus entry and transcriptional regulation (discussed above), narrowing the dynamic range of the assay and *blunting* its ability to reflect actual magnitude of cAMP oscillations. Besides, the phenomenon of signal amplification should be duly accounted for when interpreting the data from reporter gene studies—the importance of this in nicely exemplified by partial GPCR agonists, which might appear to act as full agonists (Baker et al., 2003).

As reporter assays rely on the readout from the remote point of cAMP signaling cascade, there is always a possibility of interference with other signal transduction pathways. The presence of such confounding input might significantly affect the overall results of an assay, obscuring the actual changes of cAMP in specimens under study (Hill et al., 2010). Besides, as the way from cAMP to reporter protein encompasses multiple steps, any compound or investigational molecule to be used in the assay must not interfere with all the components of normal cell machinery involved in protein synthesis (i.e., transcription, translation, protein trafficking, and post-translational modifications). In such a way, results of essentially any reporter gene-based assay might warrant further validation in at least one extra assay of different format, which is not immediately dependent on protein synthesis.

Finally, when used separately, reporter gene assays are poorly applicable for studies of cAMP spatial dynamics in various subcellular compartments, as the readout from the assays indirectly reflects only the net change in total pool of free cAMP. Hence, a signal from a reporter protein usually confers little information on the initial *hot spots* of cAMP generation and its successive intracellular flow.

To conclude, reporter gene-based assays make a reasonable choice once the transcriptional effects of cAMP are in the focus of research interest. However, if one wants to have a closer look at cAMP dynamics above the level of gene expression, e.g., patterns of cAMP synthesis, diffusion and decay, biosensors for direct cAMP probing clearly should be used instead.

## Selection of the Right cAMP Sensor for Experiment

Given the wide array of different biosensors for cAMP currently available, selection of the right one for a given experiment sometimes might present an uneasy task. Naturally, the choice of a sensor is dependent on particular research objectives. Indeed, if aiming at the levels of cAMP *per se*, direct sensors apparently make the best choice, but once the *effects* of cAMP signaling, such as transcriptional changes of cAMP-responsive genes, are in question, appropriate indirect sensors should come into action. Though the indirect sensors at large allow for the reverse scenario, i.e., reporter gene-based systems or CNGCs may be applied for measurements of cAMP oscillations, the limitations of such approach and its general inferiority to direct biosensors used for the same task should be duly recognized. Once the principle research goals have been identified, additional requirements to the readout (resolution to subcellular domains? HTS? Real-time cAMP kinetics?) should ultimately spotlight the most appropriate biosensor for the particular experimental project.

Another issue worth considering is the expertise with desired experimental techniques and availability of equipment, as some biosensors require a skillful operator and a dedicated set of tools for optimal performance (e.g., CNGCs-based sensors coupled with patch-clamp or FRET probes applied to gain subcellular resolution). Lastly, one should not disregard conventional biochemical assays for cAMP, such as ELISA, RIA, HTRF (homogeneous time resolved fluorescence) or AlphaScreen. If no high spatial or temporal resolution is required and the total cAMP levels in a sample at a single time point is all that is needed, biochemical assays for cAMP obviously are the easiest and highly reliable choice. These established analytical tools offer ready-to-use solutions with minimal requirements for optimization and are applicable to vast array of cell types and tissues, providing robust data on total cAMP levels in cellular populations (Williams, 2004; Hill et al., 2010).

**FIGURE 3:**
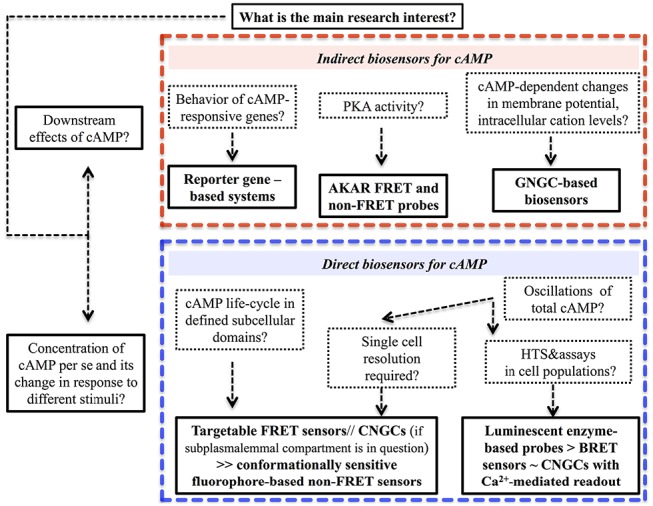
**Selection of the task-specific biosensor for cAMP probing in living cells—a decision tree**.

**FIGURE 4:**
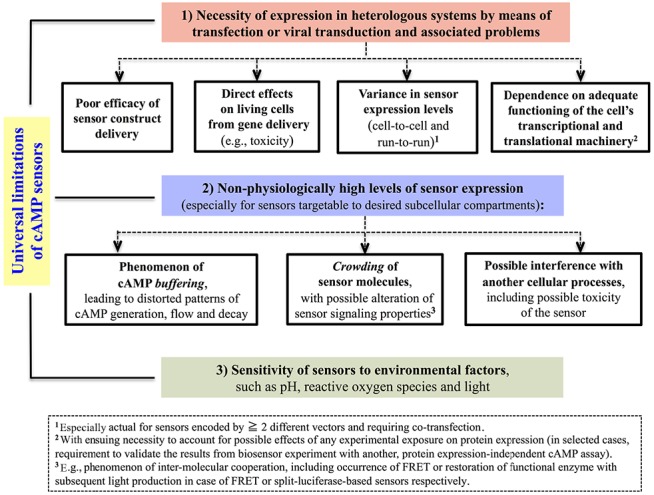
**Universal limitations inherent to most genetically encoded biosensors for cAMP**.

A decision diagram, which we hope will aid the reader in cAMP biosensor selection, is presented in Figure 3. Universal limitations, that are inherent to the bulk of the genetically encoded probes irrespectively of the nature of signal they produce, are summarized in Figure 4.

## Cutting-Edge Genetically-Encoded Tools for Modulation of cAMP in Living Systems

Apart from the biosensors reviewed earlier, recent progress in synthetic biology endowed the research community with principally novel and extremely powerful tools for modulation of cAMP in living systems. Photoactivatable adenylyl cyclases (PACs) and PDEs perhaps make the most remarkable example of such achievements. These ingenious optogenetic enzymes allow researchers to enjoy all the benefits of light as the *steering wheel*, including exquisite spatio-temporal dosing of exposure, minimal toxicity and negligible interference with physiological intercellular processes. Naturally occurring photoactivatable proteins carry light sensitive domains that translate the energy of absorbed photons into conformational rearrangements of the whole molecule. The change in protein structure triggered by light typically affects protein functional state, e.g., leading to alteration in its enzymatic activity (Karunarathne et al., 2014).

The first PAC was isolated from the unicellular flagellate *Euglena gracilis* (Schröder-Lang et al., 2007). Each subunit of this tetrameric EuPAC protein harbors a couple of photosensitive BLUF domains (Blue Light receptor Using FAD) and has own AC activity that is stimulated by blue light.

EuPAC showed excellent performance in heterologous expression systems (HEK293 cells, *Xenopus laevis* oocytes and central neural system of *Drosophila melanogaster*), with up to 10-fold enhancement of cAMP production upon exposure to blue light. AC activity of EuPAC was clearly dependent on magnitude of stimulation (both light intensity and duration of exposure) and exhibited an ultra fast on-off response within dozens of milliseconds. However, despite the remarkable functional features, EuPAC has not been widely used in successive studies, mainly due to significant dark activity and big molecular weight precluding efficient and problem-free expression.

Another PAC, discovered a few years thereafter in soil bacteria *Beggiatoa sp*. (hence the name—bPAC), did not suffer from the above limitations of EuPAC and made an extremely valuable addition to the cAMP researchers toolbox (Ryu et al., 2010; Stierl et al., 2011). This small BLUF domain-containing enzyme (350 amino acids, that is roughly one-third of the size of EuPAC) has negligible activity in the dark, which is inducible up to 300-fold upon exposure to blue light. Besides, in comparison to EuPAC this native bacterial photoenzyme requires much less irradiation to generate comparable concentrations of cAMP and preserves light-induced active conformation for longer time, thus providing more durable response. Blue light stimulates enzymatic activity of yet another PAC, identified in cyanobacterium *Microcoleus chthonoplastes* (Raffelberg et al., 2013). In contrast to the BLUF PACs discussed above, this enzyme, coined mPAC, has a LOV (Light, Oxygen, Voltage) domain for light-sensitive unit, but otherwise operates in the similar fashion.

Despite successful use of blue-light sensitive PACs *in vitro* and in selected small animal models (i.e., *Drosophila*), the general application of these tools to *in vivo* setting is hindered by low tissue penetration depth of blue light (Wan et al., 1981). PACs, activatable by near-infrared light, which can pass in bodily tissues as far as several centimeters, provide an excellent alternative. The first PAC of this kind was engineered by Ryu et al. (2014), who fused together a light-sensitive domain of phytochrome from *Rhodobacter sphaeroides* and AC from bacterium *Nostoc sp*. The resulting chimeric enzyme was efficiently regulated by red light, including *in vivo*, as was demonstrated by an alteration of locomotory activity in *Caenorhabditis elegans*, expressing this PAC in cholinergic neurons.

On the opposite end of cAMP regulation, a light-activatable phosphodiesterase (LAPD), recently introduced by Gasser et al. (2014), represents a fine-tunable instrument for cAMP degradation. A chimera protein of photosensitive phytochrome from *Deinococcus radiodurans* and human PDE2A, LAPD is an extremely versatile optogenetic enzyme, as it responds to both blue and red light with an increase of PDE activity reaching sixfold, and is down-regulated by far-red light. LAPD was already successfully tested in cell cultures and zebrafish embryos, and will probably soon be applied in larger animal models (Gasser et al., 2014).

To conclude, ACs and PDEs engineered as optogenetic tools open breath-taking avenues for cyclic nucleotide research. As genetically-encoded single chain proteins, these photoactivatable enzymes are proven to be applicable to majority of modern model systems, including *in vivo* setting. Secondly and luckily enough, the indispensable cofactors for all of the discussed optogenetic proteins, that are required for efficient sensing of photon energy and its relay to conformational changes, are naturally present in sufficient quantitates in most types of living cells and tissues (i.e., biliverdin chromophore for LAPD and red light-dependent PACs, flavins for BLUF and LOV-harboring PACs), thus obviating the need for their exogenous supply. In such a way, all that is needed for triggering the desired activity (AC or PDE) in a biological sample under study—is to illuminate it with the light of appropriate wavelength and intensity. With spatial resolution defined by the width of the light beam and ultra-flexible timing of stimulation, optogenetics enzymes are a way ahead of the pharmacological agents traditionally used in cAMP research, such as non-specific AC stimulator forskolin (FSK) and PDE inhibitor IBMX. The engineering of PACs and PDE activated by red and near-infrared light paves the way for non-invasive modulation of cAMP in living animals, allowing to study and fine-tune cyclic nucleotide signaling in otherwise non-disturbed tissue microenvironment. Moreover, one can readily envision the combined used of photoactivatable ACs and PDEs in a single cellular system or animal model for a *full cycle* cAMP control, provided that both enzymes have well-separated spectral characteristics.

Apart from the photoactivatable proteins, we would like to highlight another pair of genetically-encoded tools that may be fruitfully employed in living systems for cAMP modulation. The first instrument is based on a modified soluble AC (sAC), a special isoform of AC that is directly regulated by bicarbonate ions (HCO_3_^–^) and involved in pH sensing in a variety of tissues (Rahman et al., 2013). By decorating truncated catalytically active rat sAC with the appropriate localization signals, Sample et al. (2012) were able to express sAC constructs in desired domains of living cells. Stimulation of the cells with bicarbonate readily triggered fast and sustained activation of sAC leading to rise in cAMP in the subcellular compartments sAC was targeted to. Moreover, sAC activity was proportional to the concentration of bicarbonate anions and quickly returned to basal state after bicarbonate removal. Therefore, the engineered sACs provide quite precise and versatile means of chemical control of cAMP production, making a nice addition to PACs.

The second instrument, named *cAMP sponge*, represents a truncated variant of the RIb regulatory subunit of PKA, which preserves high affinity for cAMP, but cannot dimerize or bind PKA catalytic subunits (Lefkimmiatis et al., 2009). In such way, *sponges* are designed to act as scavengers of cAMP molecules that limit availability of free cAMP to its effectors, but do not interfere with cAMP generation or degradation. *Sponge* proteins can also be routed to certain subcellular compartment (cell membrane, cytoplasm, or cell nucleus) by means of addition of appropriate localization signals and were shown to blunt cAMP oscillations triggered by either GPCRs activation or FSK. Besides, *cAMP-sponges* successfully attenuated PKA activity, measured in living cells by AKAR sensors. These features make sponges potentially useful tools for complex experimental perturbations of cAMP signaling in different microdomains of living cells.

All in all, modern genetically encoded proteins for cAMP modulation provide hitherto unattainable opportunities for experimental interference and modeling of such major aspects of cAMP life cycle as cAMP generation, trafficking and hydrolysis. The genuine power of these tools obviously dwells in synergy with biosensors for cAMP. Flexibility of design and targetability to desired subcellular compartments, inherent to both modern probes and instruments for cAMP modulation, combined with excellent spatiotemporal resolution of selected biosensors, in principle allow for real-time studies of precisely induced cAMP waves across several domains of a single living cell. Apart from this, introduction of a *second opinion* (e.g., FRET bioprobes for Ca^2+^) shall expand research potential of a living system even further, opening avenues for insightful studies of cAMP interaction with other intracellular players. Recent results, obtained with the help of combinatorial applications involving several distinct genetically-encoded proteins, support the bright perspectives of this methodology—that is coming of age and shaping the vectors of future research endeavors (Sample et al., 2012; Tsvetanova and von Zastrow, 2014).

Currently, genetically-encoded tools for cAMP fuel the transitional phase of the whole filed of cyclic nucleotide research, shifting the focus from the unresolved and single time point measurements in cellular populations toward the pinpoint real-time modulation and probing of cAMP in discrete subcellular domains of a given cell. And with the present pace of biotechnology the newer and even more advanced instruments are quite likely to come, giving us the possibility to challenge established ideas and pursuit even bolder hypotheses.

## *A Perfect Sensor* for cAMP in Living Systems—Prospects for the Future

If we could think of a *perfect sensor for cAMP,* what principle features should it have? Firstly, and in contrast to all of the biosensors discussed in this review, a perfect sensor should be totally independent of physiological/pathophysiological processes, occurring in a living system, including transcription, translation and post-translational modifications. Obviously, in order to make this possible, a sensor has to be delivered to a system in a ready-to-use state, thus obviating the need of conversion of a gene template into a functional sensor protein by recipient cell’s protein synthesis machinery, which is the case with all genetically-encoded sensors for cAMP presently available. Only then it would be possible for a sensor to produce the most objective readout of cAMP dynamics in living cells or tissues under virtually any possible condition, including such extremes as pronounced stress or advancing cell death. In such fashion, a sensor that is based on a biological cAMP—responsive domain, might still be considered a genetically-encoded tool, but transcription and translation events from a coding sequence should happen in a dedicated expression system (e.g., bacteria, plant cells, or yeast) and not in the living system to be analyzed.

Secondly, a *perfect* sensor should be easy to deliver to living cells, with the procedure of delivery *per se* affecting cellular well-being as minimally as possible. This will also facilitate the measurements of cAMP levels in *delicate* primary cells or even tissues (*in vivo*). Targetability of a sensor to selected intracellular compartments, such as nucleus and mitochondria, would be highly desirable as well. Lastly, an ideal sensor designed for measurement of absolute values of cAMP should directly bind and sense cAMP molecules, with binding event being immediately coupled to signal generation and rapidly reversible, producing flash ON–OFF responses and minimizing cAMP buffering. Apart from the favorable kinetics of response to cAMP, allowing for real-time measurements, the signal from a sensor should be reasonably strong, providing sufficient spatial resolution for studies of cAMP oscillations in subcellular domains of single living cells.

For the time being, we could not think of any possible unimolecular sensor meeting the above criteria. The solutions might possibly lie in the interface of synthetic biology and nanotechnology, culminating in engineering of such *smart* devices, as nanoparticulate carriers equipped with cAMP-sensing elements. Nanocarriers already hold a strong position in the field of drug delivery and might make an excellent vehicle for cAMP sensors. A huge variety of different platforms, from liposomes and nanorods to silica- or metal-based nanoparticles, offers immense design flexibility—by means of decoration with various functional elements (antibodies, ligands to endogenous receptors expressed in particular cell types, *smart* bioshells, degradable by selected enzymes or in response to pH change), nanocarriers can be conceptually engineered for highly selective transportation of cAMP sensors to desired cellular population or tissue types in living multicellular organisms, let alone cell lines or primary cultures *in vitro* (Sahay et al., 2010; Sikorski et al., 2015). With the current advances in the nanoscience, we are fully justified to expect development of dedicated nanocarriers for cAMP sensor delivery in the nearest future. Of note, alternative solutions for a delivery of ready-to-use cAMP sensors might well arrive from a different direction—for instance, one could envision that a biosensor could be delivered ready-to-use by means of one of the protein transduction domains, also called cell-penetrating peptides, such as TAT, PEP-1, Antennapedia, or Arg-9 (Heitz et al., 2009). The delivery of sensors by such peptides could be coupled with a protein localization signal and even a *switch* that becomes activated only in specific cell compartments and/or cell types. This approach, to the best of our knowledge, has not been yet explored.

As for the principal component of any cAMP sensor, that is a unit responsible for binding of cAMP molecules and relaying the binding events into a traceable signal, we envision that the future generations of these elements will primarily stem from cAMP binding molecules originally evolved in nature and subsequently modified for the purpose, just like as already happened to all genetically-encoded cAMP sensors engineered so far. However, apart from the established biological cAMP effectors, such as PKA, Epac proteins and CNGCs, new *scaffolds* for cAMP sensor elements are likely to emerge, with ribozymes (RNA molecules with enzymatic activity) being a good and promising example (Koizumi et al., 1999). Combinatorial use of the above natural cAMP effectors with purely synthetic tools, e.g., cAMP-avid RNA aptamers, might lead to the creation of principally new classes of cAMP sensors with improved capabilities (Koizumi and Breaker, 2000; Paige et al., 2012; Kellenberger et al., 2013). Finally, further studies in the fundamental principles of cAMP signaling organization might well uncover hitherto unrecognized cAMP counterparts, opening new horizons for cAMP biosensors design.

## Author Contributions

VMP wrote the initial draft; VM, CS, and ARM corrected the draft and provided feedback. All authors read and approved the final version of the manuscript.

### Conflict of Interest Statement

The authors declare that the research was conducted in the absence of any commercial or financial relationships that could be construed as a potential conflict of interest.

## Abbreviations

AC: adenylyl cyclase
AKAR: A-kinase activity reporter
BLUF: blue light receptor using FAD
BRET: bioluminescence resonance energy transfer
CaM: calmodulin
cAMP: 3′-5′-cyclic adenosine monophosphate
CCD: charge-coupled device
CFP: cyan fluorescent protein
cGMP: 3′, 5′-cyclic guanosine monophosphate
CNBD: cyclic nucleotide-binding domain
CNGC: cyclic nucleotide-gated channel
CRE: cAMP-response element
CREB: cAMP-response element-binding protein
Epac: exchange protein, directly activated by cAMP
FlCRhR: FRET sensor based on PKA (Fluorescein + Catalytic unit/Rhodamine + Regulatory unit)
FRET: Förster resonance energy transfer
FSK: forskolin
GFP: green fluorescent protein
GPCR: G protein-coupled receptor
HCN: hyperpolarization-activated cyclic nucleotide-gated channels
HTS: high-throughput screening
IBMX: 3-isobutyl-1-methylxanthine
LAPD: light-activatable phosphodiesterase
LOV: light-oxygen-voltage-sensing domain
NO: nitric oxide
PAC: photoactivatable adenylyl cyclase
PDE: phosphodiesterase
PKA: protein kinase A
RIIβ: regulatory subunit IIβ of protein kinase A
Rluc: *Renilla reniformis* luciferase
sAC: soluble adenylyl cyclase
YFP: yellow fluorescent protein.
